# Production of AC from Bamboo, Orange, and Paulownia Waste—Influence of Activation Gas and Biomass Maturation

**DOI:** 10.3390/ma16093498

**Published:** 2023-05-01

**Authors:** Carlos Grima-Olmedo, Laura M. Valle-Falcones, Dulce Gómez-Limón Galindo, Ramón Rodríguez-Pons Esparver

**Affiliations:** 1 Departamento de Ingeniería Geológica y Minera, Escuela Técnica Superior de Ingenieros de Minas y Energía, Universidad Politécnica de Madrid, 28003 Madrid, Spain; lauramaria.valle@upm.es (L.M.V.-F.); dulce.gomezlimon@upm.es (D.G.-L.G.); ramon.rodripons@upm.es (R.R.-P.E.); 2 Tecminergy, Universidad Politécnica de Madrid, 28906 Getafe, Spain

**Keywords:** agricultural waste, maturity, valorization, vertical furnace, carbonization, activation

## Abstract

The production of agricultural waste is associated with environmental problems and risks to public health. The general interest demands, as an ecological alternative, the proper management of waste generated by industrial activity through its transformation into value-added products. Carbonization/activation (2 h/2 h) at 700 °C in a vertical furnace (20 K/min), to produce biochar and activated carbon (AC) from bamboo, orange, and paulownia residue, was carried out in a laboratory facility with physical activation by CO_2_ and steam. The characterization of the carbonaceous material obtained was based on the determination of the N_2_ adsorption–desorption isotherms at 77 K, the specific surface area with the BET procedure, and its internal structure by means of SEM images. The BET surface area values obtained as a function of the CO_2_/steam agent used were 911/1182 m^2^/g, 248/388 m^2^/g, and 800/1166 m^2^/g for bamboo, orange, and paulownia, respectively. The range of variation of porosity in paulownia residue generated after steam activation was 485–1166 m^2^/g, varying depending on the degree of maturity of the biomass used. Research has shown that both the type of activation agent used to produce AC and the degree of plant maturation of the precursor residue affect the quality and characteristics of the final product.

## 1. Introduction

Activated carbon (AC) is the most widely used adsorbent in industry due to its high and varied internal porosity, the ease and speed with which it retains certain pollutants, and the possibility of being reused.

AC has a reticular crystalline structure with structural units consisting of parallel layers of carbon atoms arranged in regular hexagons, forming a three-dimensional network with spaces between its layers, whose walls generate a high capacity to adsorb substances [1]. It is widely used as an adsorbent in the purification of waste streams produced by the food and beverage processing, pharmaceutical, chemical, petroleum, mining, nuclear, and automotive industries.

The production of AC from non-renewable raw materials such as coal, lignite, peat, or wood is costly and time-consuming [2]. As an ecological alternative, there is growing interest in the use of industrial by-products and agricultural waste [3,4,5] such as bamboo, paulownia, and orange peel, which generate large amounts of biomass. 

Bamboo is a hardy and versatile plant that has been used for centuries around the world for a wide variety of purposes. Paulownia is a fast-growing tree native to Asia that has achieved great popularity. Both species provide light and resistant woods suitable for reforestation and use in construction and decorative projects [6,7,8,9].

Orange peel is a solid waste produced during the processing of citrus fruit for juice production, with approximately 50–60% of the processed fruit becoming waste [10]. Historically, the most common way to dispose of this residue has been through combustion [2,11], or by using it as feed for livestock [12]. Its processing entails a number of problems, mainly due to its high organic load, the need for a disposal area, increased transportation costs, the risk of contamination of aquifers, and, in some cases, uncontrolled methane production [13].

This reality highlights the importance of seeking solutions for the treatment of agricultural residue and represents an opportunity to obtain value-added products through their transformation into biofuels, chemical products, and other sustainable materials. In this sense, its transformation into AC by means of a carbonization process in an inert atmosphere followed by physical or chemical activation provides an efficient application for a waste whose disposal entails a series of economic and environmental problems. 

The pyrolyzation or carbonization process in the absence of oxygen is carried out in the usual range of 400–900 °C to remove volatile components and considerable amounts of tar produced by the plant matter, resulting in a solid residue (biochar) with a relatively high carbon content and preliminary porosity [14]. Chemical activation requires a post-heat treatment process to recover the chemical agents, which, from an environmental point of view, may limit its application [15].

Thermal treatment of biomass is one of the main techniques for its valorization in the form of solid (biochar), liquid (bio-oil), and gaseous (syngas) products. Biochar can be obtained, among others, from agricultural, forestry, urban, and seafood waste [16], promoting its recycling and subsequent use in the removal of pollutants such as antibiotics, heavy metals, and excess nutrients from wastewater. Compared to chemical treatments for wastewater purification with flocculants, coagulants, oxidants, and microorganisms [17], adsorption with microporous materials is a more effective [18] and cheaper process [19].

Most AC applications require a second stage of activation with mild oxidizing agents, such as carbon dioxide [20,21] and steam [22,23,24,25], to create new pores, widen, clean, and connect existing ones. Using analytical techniques, it is possible to quantify the increase in carbon content, the removal of volatile components, the characteristics of the condensed liquid, the increase in porosity, and the change in the internal structure of the precursor material used.

The AC produced from biomass has been used for the adsorption and control of air pollutants, for the treatment of industrial gas emissions [26]. Due to its adsorption capacity comparable to a commercial product, it is used to reduce turbidity and the presence of COD and TDS in liquid effluent [27]. The applications of low-cost AC obtained from agricultural residue have been oriented in recent years towards the removal of pharmaceuticals [28] and for the adsorption of metal ions such as nickel [29] and uranium [30] in aqueous mixtures and solutions. Another energy application of industrial interest is the manufacture of capacitive electrodes, used in the manufacture of electrolyzers for the generation of hydrogen [31,32], and as a porous material for hydrogen storage [33].

This paper presents the possibility of reusing agricultural, forestry, and industrial waste [34,35,36] as a low-cost precursor material to produce AC, promoting the responsible use of natural resources and contributing to the promotion of the circular economy as a production and consumption model for society. 

This paper provides information on the design of a facility for the transformation of bamboo, orange peel, and paulownia plant residue into biochar and AC. The thermogravimetric characterization carried out on the precursor residue has made it possible to choose the appropriate heating temperature value within a certain range. In addition to the temperature, the activation/pyrolyzation time and the heating ramp have been established based on numerous previous research studies. The effect of the gas used (CO_2_ and steam), as well as the degree of maturation/aging of the plant biomass (P1, P3, P5, and P8) on the quality of the final charcoal obtained, has been studied.

## 2. Materials and Methods

### 2.1. Raw Material

Two lignocellulosic residues collected from the remains of bamboo cane (B) and paulownia wood of 1, 3, 5, and 8 years of growth or maturation (P1, P3, P5, and P8) were used in this work. The third residue used was obtained from the remains of orange peel (O) from citrus processing.

Manufacturer: Cotevisa (Bamboo and Paulownia)–Grupo García Carrión (Orange)

Cities: Valencia–Huelva

Country: Spain

The generation of these plant residues is associated with environmental problems such as accumulation in landfills, greenhouse gas emissions during the decomposition process, and water and soil contamination, as well as risks to public health.

**Figure 1:**
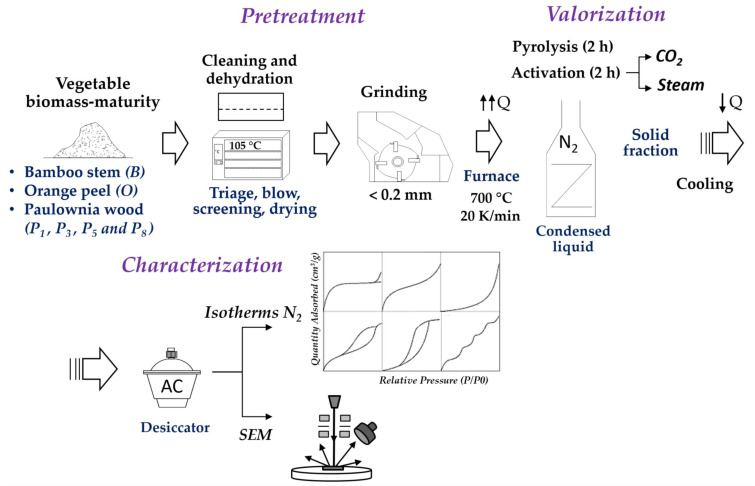
Flowchart of the plant biomass valorization process.

The development of AC from these wastes aims to seek an environmentally responsible industrial application while providing added economic value and contributing to the reduction of CO_2_ emissions. For this purpose, they will be subjected to a pyrolyzation process in an inert atmosphere with nitrogen followed by physical activation with carbon dioxide and steam to compare the development of their internal porosity. The flowchart in Figure 1 shows the process followed by the waste from collection, conditioning, recovery, and final characterization.

Soil and non-organic residue were removed from the agroforestry residue used in this work, first manually and then by sieving. The bamboo (stem), orange (epicarp), and paulownia (bark) were blown clean of dust, leaves, and branches.

**Figure 2:**
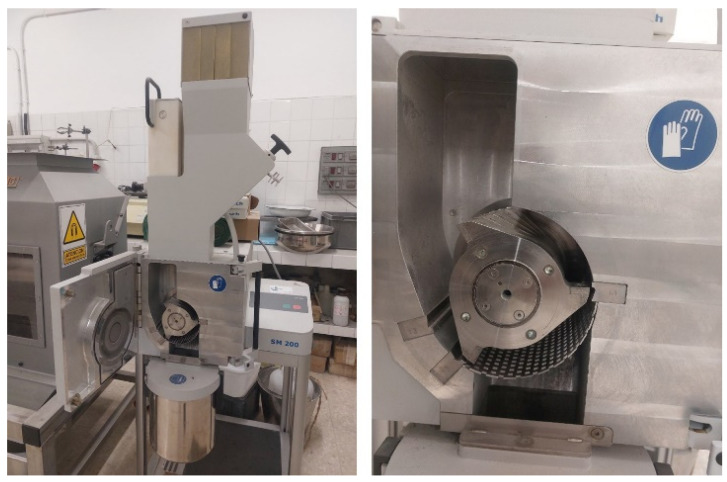
Cutting mill with bottom sieve.

They were subjected to a dehydration process at 105 °C for 10 h and were ground to increase their specific surface area in a blade mill (Retsch SM200) to a particle size of less than 0.2 mm (Figure 2).

Thermal decomposition of the three main components of lignocellulosic biomass occurs at increasing temperature ranges, hemicellulose (250–350 °C), cellulose (325–400 °C), and lignin (300–550 °C). The gases released during the thermal degradation of the lignocellulosic material are primarily CO_2_, CO, H_2_, CH_4_, C_2_H_6_, and C_2_H_4_ [37]. Cellulose and hemicellulose contribute to the bio-oil production yield, while lignin produces a higher proportion of solid carbon [38]. 

**Figure 3:**
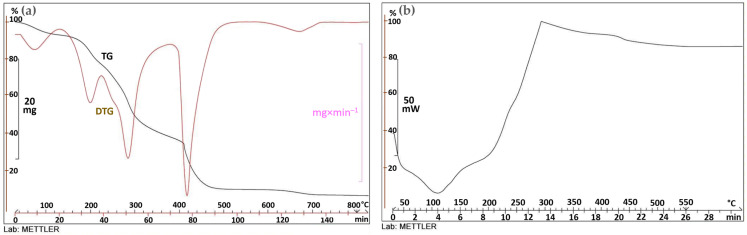
Orange peel thermogravimetric (**a**) and calorimetric analysis (**b**).

The orange epicarp is mainly composed of sugars (25.95%, volumetric titration), protein (5.44%, as 6.25×N determined by the Dumas method), ashes (6.92%), crude fiber (10.01%), moisture (9.91%), and calcium (1.78%, gravimetry). The ashes are mostly salts of organic acids that are decomposed by calcination; the remaining nutrients, not individually quantified, are fats, starch, and oils. A thermal susceptibility analysis was carried out and it showed that no exothermic reaction occurred when the sample was attacked with peroxide, indicating that it lacks any particular affinity or reactivity towards oxygen. Thermal analysis revealed the onset of slow exothermic processes at 108 °C and above, with oxidation reactions accelerating at 182 °C and net reactions at 202 °C and above (Figure 3). When heated, it produces vapors that ignite at 260 °C.

Figure 3a shows the thermogravimetric curve (TG) of the orange residue, with the red derived curve (DTG) superimposed on it. The material undergoes a multistage, stepwise, weight-loss process, which starts at 178 °C and is immediately accentuated around 182 °C. Subsequent reaction phases characterized by peaks of the derivative curve at 296 °C and 440 °C are produced. Figure 3b represents the calorimetric curve of the sample, with descending and ascending traces corresponding to endothermic and exothermic processes, respectively. The sample slowly starts an exothermic process at 108 °C, which accelerates from 202 °C and continues up to 297 °C. From this point on, the process becomes slightly endothermic until it reaches 550 °C, where the curve takes on a horizontal shape.

**Figure 4:**
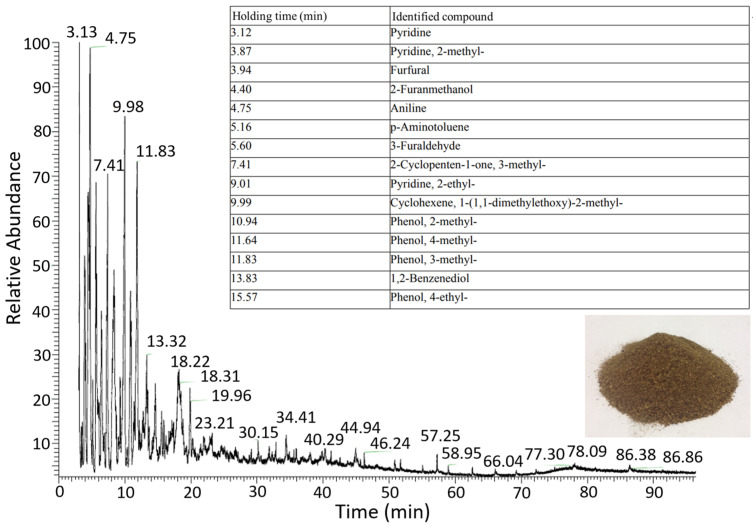
Chromatogram of condensed liquid during the carbonization process of orange residue.

The heating of the orange peel also produces an orange–brown condensed liquid containing a solid phase, deposited at the bottom of the container, and a liquid phase (bio-oil). The condensed liquid is analyzed by taking 3 mL and a liquid–liquid extraction with chloroform. The sample extracted under constant temperature and humidity conditions is passed through a 0.20 µm PTFE filter and analyzed by a gas chromatograph and ion trap mass spectrometer detection. In the chromatogram (Figure 4), the components identified were pyridine, cyclopentane, cyclohexane, aldehydes (e.g., furfural), benzenes (e.g., aniline), phenols, and aminotoluene.

### 2.2. Experimental Apparatus

The process of carbonization and physical activation of the plant residue is carried out in a laboratory facility with supply bottles, mass controllers and gas mixers, a vaporizer–cooler system, an iron–chromium alloy (kanthal) tubular reactor, and a vertical furnace.

**Figure 5:**
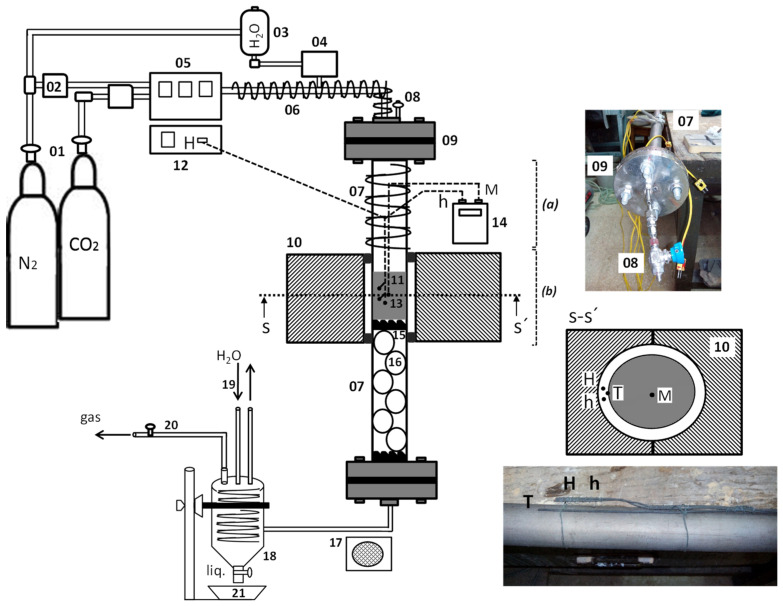
Device for pyrolyzing and activation of plant residue; 01: Gas bottle; 02: Flow controller; 03: Water tank; 04: Vaporizer; 05: Gas mixer; 06: Heating belt; 07: Tubular reactor; 08: Purge; 09: Gasket; 10: Vertical furnace; 11: Sample; 12: Furnace control; 13: Thermocouples (type K); 14: Thermometer; 15: Clamping piece; 16: Quartz balls; 17: Fan; 18: Collector container; 19: Condensation system; 20: Gas outlet; and 21: Condensate container.

Figure 5 shows in detail the scheme of the experimental carbonization device for the production of biochar in the absence of oxygen, where the volatile components are eliminated, carbon is enriched, and physical activation is performed with carbon dioxide and steam for the development of the internal porosity of the tested residue.

The N_2_ and CO_2_ used in the tests are supplied by pure gas cylinders (01) and regulated by means of mass flow controllers (02). The steam (V) is obtained by passing ultrapure water contained in a tank (03) through a vaporizer (04). 

In order to prevent the gases introduced into the circuit from condensing and wetting the sample, the inlet duct is heated with an electric resistance and heating tape (06) from the outlet of the gas mixer (05) to the furnace inlet. The tubular reactor (07) where the inert atmosphere is established has a purge connection (08) to eliminate residual air and graphite gaskets at its ends to ensure a vacuum (09). 

The control of the furnace temperature (10) and the heating ramp applied to the material sample (11) is carried out by the furnace controller (12). At the height of the sample on the outside of the tubular reactor, three thermocouples (13) are located, with one in contact with the kanthal surface and the other two slightly separated (section S-S’). The thermocouple T indicates the temperature of the outer surface of the tubular reactor, while the thermocouples H and h, which are slightly separated from the surface, with lower thermal inertia, are used to control the temperature rise of the furnace (electrically heated). The furnace programming allows the setting of the heating temperature at any time, with it being controlled by the thermocouple M located in the center of the sample and connected to a digital thermometer (14). 

The solid carbonaceous sample is placed in the central inner zone of the tubular reactor and held by a slotted piece (15), with a small amount of quartz fiber in its upper zone and porcelain balls up to the bottom of the tubular reactor (16), thus allowing the circulation of gases up to the exit through the lower zone. At the reactor outlet, there is a fan (17), a collection vessel (18), a condensation system (19), a gas outlet pipe (20), and a condensate collection vessel (21).

**Figure 6:**
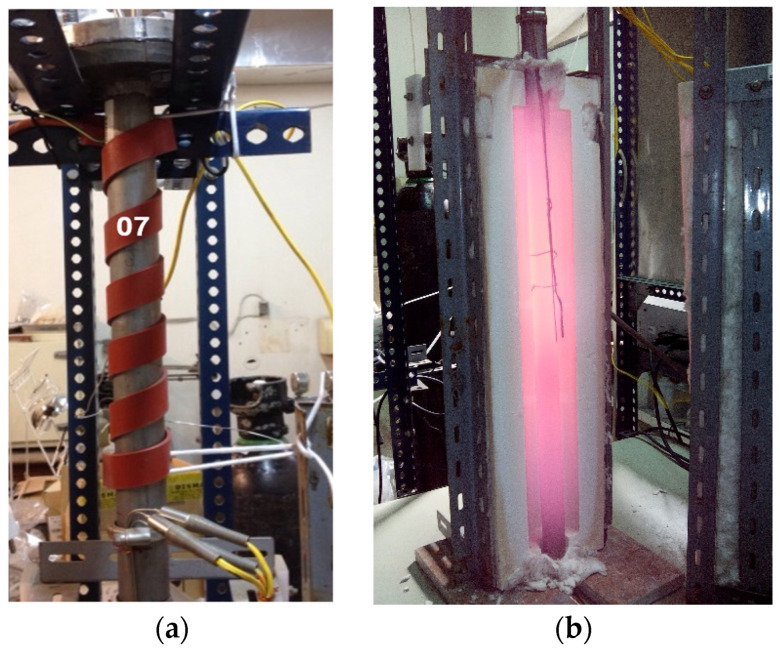
Tubular reactor (**a**) upper gas inlet area with heating tape, and vertical furnace (**b**) central zone reactor furnace with the sample housed inside it (Figure 5).

Figure 6 shows photos of the upper zone of the preheated tubular reactor with heating tape, inside which the inert atmosphere is generated with N_2_, as well as the central zone between the furnace plates where the sample is placed for pyrolyzation and physical activation.

Figure 6a shows in detail the upper part of the vertical tubular reactor through which the inertization, pyrolyzation, and activation gases are introduced to reach the sample located in the central zone of the furnace. In the photograph, inside the vertical furnace in Figure 6b, the temperature reached by the tubular reactor where the sample is housed and the location of the external thermocouples to control the heating process can be seen.

### 2.3. Pyrolysis and Activation

The process to elaborate AC starts with the pyrolyzation stage, dehydrating and devolatilizing the sample. During this stage, there is a risk of overheating due to the exothermic process or even complete combustion of the carbon in the original material. To avoid this, a previous flow of 0.5 L/min of N_2_ is passed for 30 min, eliminating the presence of oxygen in the reactor and in the sample.

The heating rate for thermal decomposition of the biomass determines the final product obtained. A high heating ramp allows higher bio-oil yields to be achieved [39], while as the heating ramp is reduced and the sample is subjected to high temperatures, secondary reactions occur [40,41] that generate a greater amount of biochar.

The internal temperature of the furnace is raised with a heating ramp of 20 K/min, until the maximum programmed set point temperature (700 °C) is reached. A flow of N_2_ is injected to maintain the inert atmosphere of the sample consisting of 10 g of bamboo, orange, or paulownia residue throughout the entire test.

In the activation stage, with the furnace at the maximum set temperature, N_2_ is replaced by CO_2_ or steam, with the latter obtained by passing ultrapure water contained in a tank through a vaporizer (Figure 5). These activating agents generate voids by selectively removing carbon atoms from the structure according to the stoichiometric reactions shown in Equations (1) and (2):

(1) $$C+{CO}_{2}\to 2CO$$

(2) $$C+H_{2}O\to CO+H_{2}$$

Excess steam when used as an activation agent generates carbon dioxide in the products of combustion shown in Equation (3):

(3) $$CO+H_{2}O\to {CO}_{2}+H_{2}$$

Activation with CO_2_ and steam produces endothermic reactions of 159 kJ/mol and 117 kJ/mol, respectively, which have easily controlled kinetics. It cannot be performed in the presence of oxygen (air), unless very low partial pressures are used, since the carbon–oxygen reaction, being highly exothermic, would cause ignition of the material without improving the porosity results.

In an endothermic carbonization process, as opposed to an exothermic one, it is easier to control the transformation rate of the sample by means of the heat applied and the residence time in the furnace in the presence of an inert atmosphere. The activation gas flow rates used were 150 mL/min for CO_2_ (approximately 0.402 mol/h) and 4.8 g/h for steam (approximately 0.267 mol/h), with this difference due to the fact that steam is more reactive than CO_2_. Once the programmed period has elapsed, the activation gas flow is cut off and the N_2_ flow is injected again, and with the furnace turned off, the sample is allowed to cool down to room temperature. Finally, the resulting material is stored in a desiccator for the corresponding characterization analyses.

Yield is the percentage of the amount of AC produced in relation to the amount of feedstock used in the pyrolysis process and is calculated on a dry basis according to Equation (4).

(4) $$Y\left(\%\right)=\frac{Activated\;carbon\;mass}{Precursor\;residue\;mass}\times 100$$

The burn-off or percentage of solid residue removed during the carbonization and activation process increases with temperature and residence time in the reactor. It is calculated with Equation (5) using analytical techniques such as elemental combustion (CHN), atomic absorption spectroscopy, or infrared spectroscopy (FTIR).

(5) $$Bo\left(\%\right)=\frac{Precursor\;residue\;mass-Activated\;carbon\;mass}{Precursor\;residue\;mass}\times 100$$

At higher burn-off, the volume of micropores and mesopores increases, but if it exceeds 70%, the process generally begins to lose microporosity [42].

The characterization of the AC obtained with respect to yield, pore size distribution, and porosity development indicates the efficiency of the process followed during pyrolyzation and activation.

## 3. Results and Discussion

### 3.1. Analysis and Performance

Table 1 shows the elemental analysis of the three dried vegetable residues and the ultimate analysis of the material obtained after the carbonization/activation processes (2 h/2 h) with steam at a temperature of 700 °C. For the activated carbons (AC), the C and N contents increased in the ranges of 59.6–78.6% and 130–240%, respectively. On the contrary, there was a decrease in H and O contents within the ranges 79.9–85.0% and 40.7–73.8%, respectively.

The results of the test with three replicates (*n* = 3) correspond to yield/burn-offs (Equations (4) and (5)) of 34.6% (±/0.32)/65.4%, 22.6% (±/0.47)/77.4%, and 31.3% (±/0.38)/68.7%, respectively, for the carbonaceous product obtained from bamboo, orange, and paulownia precursor residue.

### 3.2. Pore Size Distribution

The textural characterization of the samples is carried out by determining the N_2_ adsorption–desorption isotherms at 77 K on a Micromeritics ASAP 2020 automatic analyzer. Before analysis, the residues are degassed at 90 °C for 4 h. The information obtained is used to adjust the Langmuir equation that describes the relationship between pressure (P/P0) and the amount of adsorbed nitrogen.

**Figure 7:**
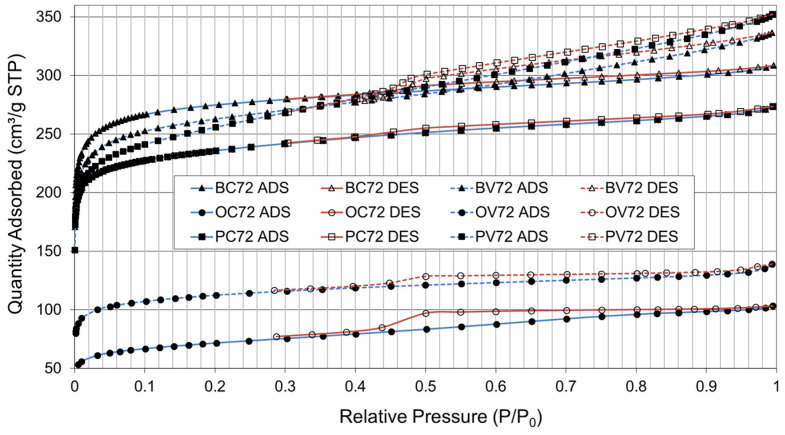
N_2_ adsorption (ADS) and desorption (DES) isotherms. Bamboo (B), orange (O), and paulownia (P) residue; activation gas: CO_2_ (C) and steam (V); temperature: 700 °C; pyrolyzation/activation time: 2 h/2 h.

Figure 7 shows the N_2_ isotherms of the AC obtained after the pyrolyzation and physical activation stages using CO_2_ (C) and steam (V), at 700 °C for 2 h each, using bamboo, orange, and paulownia residue as precursor material (maturity grade P3).

The shape of the isotherms obtained for the three residues fall into types I and IV according to the IUPAC classification corresponding to materials with microporosity (<25 Å) and mesoporosity (25–500 Å). The presence of a more developed hysteresis cycle is associated with capillary condensation occurring in its mesopores, and it limits absorption in the high-pressure range. The initial part of the type IV isotherm is attributed to monolayer formation in multiple adsorption layers as it follows the same trajectory as the type II adsorption isotherm. 

**Figure 8:**
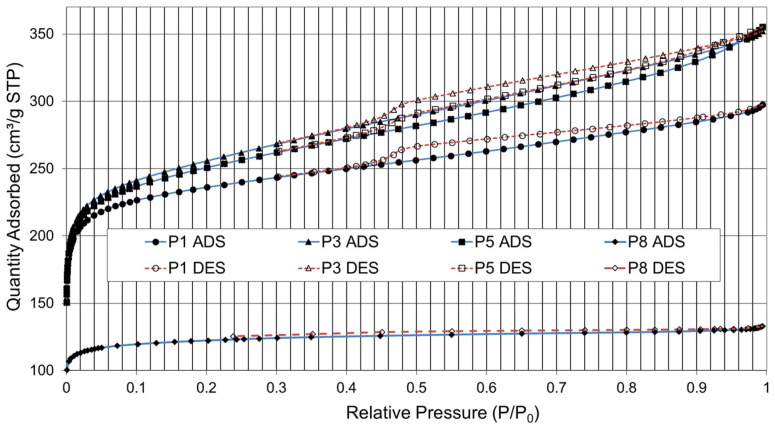
Isotherms of N_2_ Adsorption (ADS) and Desorption (DES). Residue of paulownia (P); years of maturation (1, 3, 5, and 8).

Additional tests have been conducted to determine whether the degree of maturation of the plant biomass can affect the quality of the AC produced. Figure 8 shows the adsorption–desorption isotherms obtained for four samples of paulownia, P1, P3, P5, and P8, from the same species with 1, 3, 5, and 8 years of plant development.

The porous structure of the AC obtained may vary depending on the degree of aging of the precursor. As the precursor ages, the cell walls become stiffer and denser. The presence of a horizontal plateau in the isotherm and a barely visible hysteresis cycle indicates that the solid surface is relatively homogeneous and that N_2_ molecules adsorb and desorb easily. This indicates that the solid surface has not developed a significant porous structure. 

The best nitrogen adsorption results (approx. 360 cm^3^/g) were obtained with paulownia residue that had a maturity grade of 3 years (P3). There is a decrease in charcoal quality as the biomass nears the end of its life cycle (harvesting), with a lower development in 8-year-old paulownia (P8) of its pore structure, BET area, mesopore volume, and external area. In this case, the shape of the isotherms (type I) shows a horizontal plateau and a barely visible hysteresis cycle. The initial steepest part represents the filling of the micropores and the plateau followed by a small slope is indicative of multilayer adsorption on the external surface area. The micropore filling process occurs at low relative pressures, which indicates a narrow micropore size distribution with no contribution from larger pores.

### 3.3. Surface Area and Pore Volume

The information obtained from the graphs (Figure 7 and Figure 8) is based on the hypothesis that adsorption occurs at specific sites on the surface of the material, with each site being available to a single particle of the adsorbable compound. From these settings, the BET (Brunauer–Emmett–Teller) surface area and other parameters related to the porosity of the material can be calculated. The end result is a measure of the specific surface area of the carbon, which reflects the amount of surface area available for adsorption of compounds. This information is useful, for example, to evaluate the effectiveness of AC as an adsorbent material and to develop applications in areas such as water purification and air treatment.

ACs have typical surface areas and pore volumes of 250–2500 m^2^/g and 0.022–91.4 cm^3^/g, respectively [43]. The most adsorption occurs through the micropores, with mesopores and macropores being important to allow rapid passage of adsorbed molecules to the smaller pores located internally [44]. 

This work follows a physical activation process involving carbonization and subsequent activation of the resulting charcoal in the presence of oxidizing agents such as CO_2_ and steam. Chemical activation, on the other hand, usually consists of a single step for the preparation of activated carbon in the presence of chemical agents. Chemical activation generally requires lower temperatures and shorter times for material activation. Carbon yields obtained by chemical activation are higher than those obtained by physical activation. However, it requires a post-heat treatment process to recover the chemical agents, which limits its application due to environmental concerns.

Table 2 shows the porosity results obtained after the pyrolyzation and activation stages carried out consecutively at 700 °C for 2 h for the three plant residues, using CO_2_ and steam.

The results of AC production using CO_2_ and steam as activation agents show differences in efficiency and quality of the final material. Steam, being an oxidizing agent, can react better with the components of the raw material to produce volatile compounds and increase its porosity. In addition, it has a smaller molecular size than CO_2_ and can diffuse more rapidly and access the internal structure of the material more easily. 

Table 3 shows the variables and results obtained in other previous work to produce AC by physical or chemical activation, carried out with orange (O), bamboo (B), and Paulownia (P) residue (R).

There are studies that report on the physical and chemical variation experienced by the biomass according to its degree of maturation and aging [59,60], but not how it influences the adsorptive properties of the charcoal obtained after the pyrolyzation and physical activation process. 

Table 4 shows the parameters that characterize the surface and volume of the pores generated in the steam-activated paulownia residue according to the degree of maturity of 1, 3, 5, and 8 years.

The results indicate that the best quality of AC obtained from paulownia remains with an average degree of maturity around 3–5 years, with a sharp decline when it is in its final harvesting period (8 years).

### 3.4. Particle Morphology Characterization

**Figure 9:**
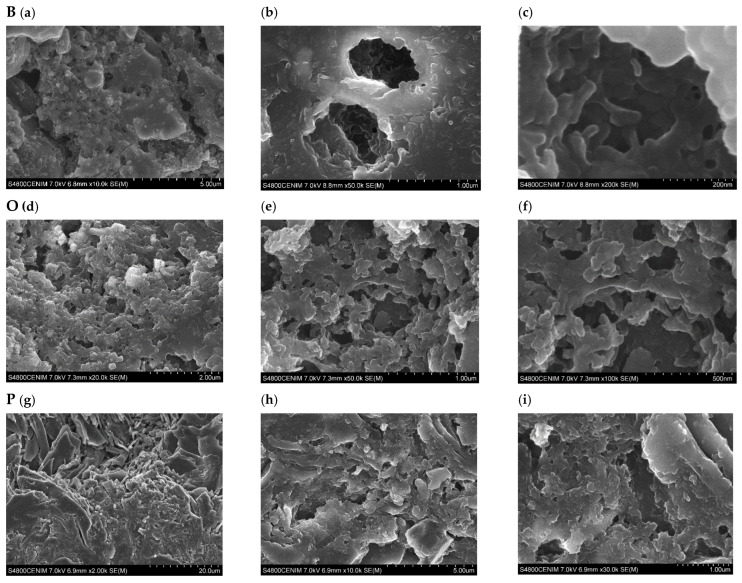
SEM images of ACs produced from bamboo B (**a**–**c**), orange O (**d**–**f**), and paulownia P (**g**–**i**) residue with a magnification factor of 10.0 k (**a**), 50.0 k (**b**), and 200 k (**c**); 20.0 k (**d**), 50.0 k (**e**), and 100 k (**f**); 2.00 k (**g**), 10.0 k (**h**), and 30.0 k (**i**).

The morphology of the developed AC depends significantly on the precursor material. Figure 9 shows SEM photographs of the internal structure of the charcoals obtained from the three plant residues bamboo (B), orange (O), and paulownia (P). Bamboo has an external surface with a sponge-like development and pore volumes with cavities connected in the form of tubes (Figure 9a–c). In the orange peel, although pores of sufficient diameter have formed, they have not achieved a development and connection that favors the increase of internal volume (Figure 9d–f). The surface of the carbonaceous paulownia material has a more heterogeneous structure with irregular channels (Figure 9g–i).

Bamboo cane is composed of long, thin fibers bound by a matrix of lignin and hemicellulose. Thermal decomposition and physical activation of cellulose and hemicellulose leads to the development and connection of porosity in the form of tubes. The presence of phenolic compounds and organic acids in orange peel may have influenced the reduced development and connection of the pores obtained with the thermal decomposition of cellulose and hemicellulose. During the physical activation of paulownia, its cell walls dilate, creating small cavities in its surface.

## 4. Conclusions and Future Directions

The experimental data and results obtained in this work may be useful for the production of AC from vegetable waste and for other researchers to establish a reliable methodology for treatment and valorization.

The AC produced from the plant biomass of bamboo cane, orange peel, and paulownia bark, using steam as the physical activation agent, developed a higher specific surface area and adsorption capacity than that produced with CO_2_. 

It is believed that CO_2_ reacts mainly with the active sites located in the center of the pores, creating micropores, and only attacks the pore walls when the activation time becomes longer [37]. Steam simultaneously modifies the active sites located both in the center and on the walls of the pores. Steam has a smaller molecular size than CO_2_ and can diffuse faster through the pore network and gain easier access to existing micropores [61,62]. Steam generally produces AC with wider pore size distributions and larger surface areas, while CO_2_ is more selective toward creating micropores. Therefore, and in agreement with other research, steam is considered to be more reactive than CO_2_ under analogous conditions [61,63].

Relative mass ratios of organic and inorganic components vary with biomass type, growth environment, and harvesting time. The quality of the AC produced is influenced by the age and degree of maturation of the plant biomass of the recovered waste. Chemical degradation and alteration processes due to aging modify the properties and composition of the organic material. Cellulose and hemicellulose molecules undergo degradation and depolymerization processes, which can reduce the carbohydrate content of the biomass and increase the proportion of lignin and other compounds more resistant to carbonization and activation.

This research has shown that both the type of activation agent used to produce AC and the degree of plant maturation of the precursor waste affect the quality and characteristics of the final product, and it is important to consider these factors in the design and optimization of the valorization processes.

The differences in surface properties obtained depending on the degree of maturation of the plant biomass point to the need to extend this study to a larger quantity of residue and their applications as low-cost adsorbents.

Machine learning is an artificial intelligence technique that involves the creation of statistical models and algorithms that can learn and improve through the analysis of large amounts of data. They can be used for the improvement of the characteristics of the obtained biochar with respect to its environmental applications [64], and to predict the yield and quality of the AC as a function of the nature of the precursor material and the conditions of the pyrolyzation/activation process [65].

Future research should focus on exergoenviroeconomic analysis to assess the energy consumption, energy efficiency, and environmental impact [66] of the production process and use as an absorbent material for AC [67]. 

The amount and type of solid waste produced and liquid effluents generated, as well as emissions of greenhouse gases and other pollutants, depend to a large extent on the precursor material and the production process used. In addition, the environmental impact associated with the extraction and transportation of raw materials should be evaluated in comparison with the use of agricultural and forestry waste, etc. 

Finally, the analysis of the energy used for the evaluation of systems and processes [68] can provide a more complete view of the sustainability and energy efficiency of the waste valorization process studied.

## Figures and Tables

**Table 1:** Elemental analysis of the precursor materials and ultimate analysis of activated carbons.

R→AC	C (%)	H (%)	N (%)	S (%)	O (%) by Difference
B→BV	46.60→78.24	6.14→0.92	0.20→0.62	0.08→0.23	46.98→19.99
O→OV	41.62→66.43	5.78→1.16	0.74→1.68	0→0	51.86→30.73
P→PV	48.25→86.18	6.35→1.19	0.21→0.72	0.07→0.09	45.12→11.82

R: dry precursor residue, AC: activated carbon, B: bamboo cane, O: orange peel, P: paulownia wood, V: activated with steam.

**Table 2:** Surface area and pore size of bamboo (B), orange (O), and paulownia (P) residue, pyrolyzed/activated at 700 °C (2 h/2 h) with CO_2_ (C) and steam (V).

Residue (Precursor)		Bamboo (Stem)		Orange (Epicarp)		Paulownia (Bark)	
Activated carbon (AC)	Unit.	BC	BV	OC	OV	PC	PV
BET surface area	m^2^/g	911	1182	248	388	800	1166
Micropore area	m^2^/g	855	1043	175	351	734	967
	%	93.85	88.19	70.39	90.41	91.75	82.94
Total vol. BJH	cm^3^/g	0.477	0.520	0.160	0.215	0.423	0.545
Micropore volume	cm^3^/g	0.407	0.349	0.081	0.163	0.345	0.318
	%	85.26	67.07	50.79	75.73	81.63	58.46
Average pore diameter	nm	3.22	4.32	3.79	3.36	2.77	3.96

B: bamboo, O: orange, P: paulownia. Activated with C: CO_2_, V: steam.

**Table 3:** AC obtained by physical or chemical activation.

Physical Activation						
R	Gas	Flow (mL/min)	Temperature (°C)	Time (h)	BET (m^2^/g)	Ref.
O	N_2_ (Py)	150 cm^3^/min	700	2	20	[45]
	CO_2_ (Ac)	150 cm^3^/min	700	2	511	
	N_2_ (Py)	150 mL/min	700	2	-	[46]
	CO_2_ (Ac)	60 mL/min	725–800	-	405–680	
	N_2_ (Py)	-	500–1200	1	-	[47]
	CO_2_ (Ac)	-	500–1200	1	225.6–248	
B	N_2_ (Py)	300 cm^3^/min	900	1	-	[48]
	CO_2_ (Ac)	-	900	0.5–1	391	
	N_2_ (Py)	500 mL/min	800	-	-	[49]
	Steam (Ac)	400 mL/h	800	1	464.7–589.6	
	N_2_ (Py)	500 mL/min	800–900	1–2	-	[50]
	Steam (Ac)	400 mL/h	800–900	2	474–794	
	CO_2_ (Ac)	400 mL/min	800–900	2	570–594	
Chemical activation						
R	Gas	Flow (mL/min)	Temperature (°C)	Time (h)	BET (m^2^/g)	Ref.
O	H_3_PO_4_	2:1	475	0.5	1090	[51]
	Ar_2_ + KOH	30 cm^3^/min +2:5–8:5	550	4	676–897	[52]
	Ar_2_ + KOH	30 cm^3^/min +8:5	550	4	625	[53]
B	H_3_PO_4_	1:1	600	0.5–1	1215–1416	[54]
	H_3_PO_4_	1:1–6:1	600–900	4	697–2123	[55]
	K_2_CO_4_	1:2–2:1	500–900	1	631–2175	[48]
P	N_2_(P)	-	600	2	-	[56]
	KOH + HCl	2:1–4:1	800	1	1006–1471	
	KOH + HCl	2:1–6:1	600–900	0.5–2	782–2435	[57]
	ZnCl_2_ + HCl	1:2–4:1	400–700	1	1264–2620	[58]

R: precursor residue, B: bamboo, O: orange, P: paulownia.

**Table 4:** Textural parameters of AC obtained from paulownia residue with different degrees of maturity, pyrolyzed/activated at 700 °C (2 h/2 h) with steam.

Paulownia Maturity	Years	1	3	5	8
Activated carbon (AC)	Unit.	P1	P3	P5	P8
BET surface area	m^2^/g	1063	1166	1143	485
Micropore area ^1^	m^2^/g	943	967	958	430
External surface area ^1^	m^2^/g	121	199	185	55
Micropore volume ^1^	cm^3^/g	0.318	0.318	0.315	0.165
Average pore diameter ^2^	Å				
Adsorption		38.85	39.57	43.74	3.13
Desorption		49.61	49.96	56.73	4.23
Average pore hydraulic radius ^3^	Å	3.43	4.07	4.04	0.51

Calculation method: ^1^ t-Plot, ^2^ BJH (4 V/A), ^3^ MP-Method (V/A).

## Data Availability

Not applicable.

## Abbreviations

AC	Activated carbon
ADS	Adsorption
BET	Brunauer–Emmett–Teller equation
BJH	Barrett, Joyner, and Halenda theory
COD	Chemical Oxygen Demand
DES	Desorption
DTG	Derivative TG
PTFE	Polytetrafluoroethylene
SEM	Scanning Electron Microscope
TDS	Total Dissolved Solids
TG	Thermogravimetric curve
Internal Notation	
Ac	Activation
B	Bamboo stem
Bo	Burn-off
C	Activation gas CO_2_
O	Orange peel
P	Paulownia wood
P 1,3,5,8	P with 1, 3, 5, and 8 years maturation
Py	Pyrolyzation
R	Precursor residue
V	Activation steam
Y	Yield

## References

[1] Rodríguez-Reinoso F., Molina-Sabio M. (1998). Textural and chemical characterization of microporous carbons. Adv. Colloid Interface.

[2] Lapuerta M., Hernandez J., Pazo A., Lopez J. (2008). Gasification and co-gasification of biomass wastes: Effect of the biomass origin and the gasifier operating conditions. Fuel Proc. Technol..

[3] Devarly P., Kartika Y., Indraswati N., Ismadji S. (2008). Activated carbon from jackfruit peel waste by H3PO4 chemical activation: Pore structure and surface chemistry characterization. Chem. Eng. J..

[4] Halbus A.F., Athab Z.H., Hussein F.H. (2021). Review on preparation and characterization of activated carbon from low cost waste materials. Egypt. J. Chem..

[5] Somparn W., Panyoyai N., Khamdaeng T., Tippayawong N., Tantikul S., Wongsiriamnuay T. (2020). Effect of process conditions on properties of biochar from agricultural residues. IOP Conf. Ser. Earth Environ. Sci..

[6] Dubinin M.M., Plavnik G.M., Zaverina E.D. (1964). Integrated study of the porous structure of active carbons from carbonized sucrose. Carbon.

[7] Ikram M., Zahoor M., Batiha G.E.-S. (2021). Biodegradation and decolorization of textile dyes by bacterial strains: A biological approach for wastewater treatment. Z. Für Phys. Chem..

[8] Khayam S., Zahoor M., Khan E., Shah M. (2020). Reduction of keto group in drimarene blue by aspergillus niger: A predominant reason for subsequent decolorization. Fresen. Environ. Bull..

[9] Ikram M., Zahoor M., Khan E., Khayam S. (2020). Biodegradation of Novacron Turqueiose (Reactive Blue 21) by Pseudomonas aeruginosa. J. Chem. Soc. Pak..

[10] Wilkins M.R., Suryawati L., Maness N.O., Chrz D. (2007). Ethanol production by Saccharomyces cerevisiae and Kluyveromyces marxianus in the presence of orange-peel oil. World J. Microb. Biot..

[11] Ghani W.A., Alias A.B., Savory R.M., Cliffe K.R. (2009). Co-combustion of agricultural residues with coal in a fluidized bed combustor. Waste Manag..

[12] Tripodo M.M., Lanuzza F., Micali G., Coppolino R., Nucita F. (2004). Citrus waste recovery: A new environmentally friendly procedure to obtain animal feed. Bioresource Technol..

[13] Lopez J.A.S., Li Q., Thompson I.P. (2010). Biorefinery of waste orange peel. Crc. Cr. Rev. Biotechn..

[14] Sing K.S.W., Everett D.H., Haul R.A.W., Moscou L., Pierotti R.A., Rouquerol J., Siemieniewska T. (1985). Reporting physisorption data for gas/solid systems with special reference to the determination of surface area and porosity. Pure Appl. Chem..

[15] Ahmadpour A., Do D.D. (1997). The preparation of activated carbon from macadamia nutshell by chemical activation. Carbon.

[16] Adibah W., Waiho K., Azwar E., Fazhan H., Peng W., Dahlianis S., Tabatabaei M., Nai Yuh P., Almomani F., Aghbashlo M. (2022). A state-of-the-art review on producing engineered biochar from shellfish waste and its application in aquaculture wastewater treatment. Chemosphere.

[17] Pokhrel D., Viraraghavan T. (2004). Treatment of pulp and paper mill wastewater—A review. Sci. Total Environ..

[18] Dąbrowski A. (2001). Adsorption—from theory to practice. Adv. Colloid Interface Sci..

[19] Alam S., Khan M.S., Bibi W., Zekker I., Burlakovs J., Ghangrekar M.M., Bhowmick G.D., Kallistova A., Pimenov N., Zahoor M. (2021). Preparation of Activated Carbon from the Wood of Paulownia tomentosa as an Efficient Adsorbent for the Removal of Acid Red 4 and Methylene Blue Present in Wastewater. Water.

[20] Vazquez-Santos M.B., Martinez-Alonso A., Tascon J.M.D. (2012). Effects of phosphoric acid as additive in the preparation of activated carbon fibers from poly (p-phenylene benzobisoxazole) by carbon dioxide activation. J. Anal. Appl. Pyrol..

[21] Alcaniz-Monge J., Perez-Cadenas M., Marco-Lozar J.P. (2012). Removal of harmful volatile organic compounds on activated carbon fibres prepared by steam or carbon dioxide activation. J. P. Adsorpt. Sci. Technol..

[22] Wang R., Amano Y., Machida M. (2013). Surface properties and water vapor adsorption-desorption characteristics of bamboo-based activated carbon. J. Anal Appl. Pyrol..

[23] Mopoung S., Dejang N. (2021). Activated carbon preparation from eucalyptus wood chips using continuous carbonization-steam activation process in a batch intermittent rotary kiln. Sci. Rep..

[24] Budinova T., Gergova K., Petrov N., Minkova V. (1994). A study of the process of pyrolysis in a water-vapor stream of activated carbons, prepared from agricultural by-products by some physicochemical methods. Thermochim. Acta.

[25] Gergova K., Galushko A., Petrov N., Minkova V. (1992). Investigation of the porous structure of activated carbons prepared by pyrolysis of agricultural by-products in a stream of water-vapor. Carbon.

[26] Tadda M.A., Ahsan A., Shitu A., ElSergany M., Arunkumar T., Bipin J., Razzaque M.A., Nik Daud N.N. (2016). A review on activated carbon: Process, application and prospects. J. Adv. Civ. Eng. Pract. Res..

[27] Hirunpraditkoon S., Tunthong N., Ruangchai A., Nuithitikul K. (2011). Adsorption Capacities of Activated Carbons Prepared from Bamboo by KOH Activation. World Acad. Sci. Eng. Technol. Int. J. Chem. Mol. Eng..

[28] Malesic-Eleftheriadou N., Liakos E.V., Evgenidou E., Kyzas G.Z., Bikiaris D.N., Lambropoulou D.A. (2022). Low-cost agricultural wastes (orange peels) for the synthesis and characterization of activated carbon biosorbents in the removal of pharmaceuticals in multi-component mixtures from aqueous matrices. J. Mol. Liq..

[29] Herrera-Barros A., Bitar-Castro N., Villabona-Ortíz Á., Tejada-Tovar C., González-Delgado Á.D. (2020). Nickel adsorption from aqueous solution using lemon peel biomass chemically modified with TiO2 nanoparticles. Sustain. Chem. Pharm..

[30] Ying D., Hong P., Jiali F., Qinqin T., Yuhui L., Youqun W., Zhibin Z., Xiaohong C., Yunhai L. (2020). Removal of uranium using MnO_2_/orange peel biochar composite prepared by activation and in-situ deposit in a single step. Biomass Bioenergy.

[31] Forouzandeh P., Kumaravel V., Suresh P. (2020). Electrode Materials for Supercapacitors: A Review of Recent Advances. Catalysts.

[32] Zhang L., Zhao X.S. (2009). Carbon-based materials as supercapacitor electrodes. Chem. Soc. Rev..

[33] Sevilla M., Mokaya R. (2014). Energy storage applications of activated carbons: Supercapacitors and hydrogenstorage. Energy Environ. Sci..

[34] Namasivayam C., Kavitha D. (2002). Removal of Congo Red from Water by Adsorption onto Activated Carbon Prepared from Coir Pith, an Agricultural Solid Waste. Dye. Pigment..

[35] Auta M., Hameed B.H. (2011). Optimized waste tea activated carbon for adsorption of Methylene Blue and Acid Blue 29 dyes using response surface methodology. Chem. Eng. J..

[36] Davila P.A., Torres-Rivera O.L., Ramos R.L., Perez R.O. (2012). Removal of Pyridine from Aqueous Solution by Adsorption on an Activated Carbon Cloth. Clean-Soil, Air, Water.

[37] Snajdarek L., Chylek R., Pospfsil J. (2022). Slow thermal decomposition of lignocelluloses compared to numerical model: Fine particle emission, gaseous products analysis. Energy.

[38] Kan T., Strezov V., Evans T.J. (2016). Lignocellulosic biomass pyrolysis: A review of product properties and effects of pyrolysis parameters. Renew. Sustain. Energy Rev..

[39] Demirbas A., Arin G. (2002). An overview of Biomass pyrolysis. Energ. Source.

[40] White J.E., Catallo W.J., Legendre B.L. (2011). Biomass pyrolysis kinetics: A comparative critical review with relevant agricultural residue case studies. J. Anal. Appl. Pyrol..

[41] Williams P.T., Besler S. (1996). The influence of temperature and heating rate on the pyrolysis of biomass. Renew. Energ..

[42] Marsh H., Rodríguez-Reinoso F. (2006). Activated Carbon.

[43] Grima-Olmedo C., Ramírez-Gómez Á., Gómez-Limón D., Clemente-Jul C. (2016). Activated carbon from flash pyrolysis of eucalyptus residue. Heliyon.

[44] Ioannidou A.Z. (2007). Agricultural residues as precursors for activated carbon production-A review. Renew Sust. Energ. Rev..

[45] Rosas J., Bedia J., Rodríguez-Mirasol J., Cordero T. (2010). On the preparation and characterization of chars and activated carbons from orange skin. Fuel Process. Technol..

[46] Marquez-Montesinos F., Cordero T., Rodríguez-Mirasol J., Rodríguez J.J. (2002). CO_2_ and steam gasification of a grapefruit skin char. Fuel.

[47] Hashemian S., Salari K., Yazdi Z.A. (2014). Preparation of activated carbon from agricultural wastes (almond shell and orange peel) for adsorption of 2-pic from aqueous solution. J. Ind. Eng. Chem..

[48] Horikawa T., Kitakaze Y., Sekida T., Hayashi J., Katoh M. (2010). Characteristics and humidity control capacity of activated carbon from bamboo. Bioresour. Technol..

[49] Lo S.-F., Wang S.-Y., Tsai M.-J., Lin L.-D. (2012). Adsorption capacity and removal efficiency of heavy metal ions by Moso and Ma bamboo activated carbons. Chem. Eng. Res. Des..

[50] Wang S.-Y., Tsai M.-H., Lo S.-F. (2008). Effects of manufacturing conditions on the adsorption capacity of heavy metal ions by Makino bamboo charcoal. Bioresour. Technol..

[51] Fernandez M.E., Nunell G.V., Bonelli P.R., Cukierman A.L. (2014). Activated carbon developed from orange peels: Batch and dynamic competitive adsorption of basic dyes. Ind. Crops Prod..

[52] Moreno-Piraján J.C., Giraldo L. (2012). Heavy metal ions adsorption from wastewater using activated carbon from orange peel. E-J. Chem..

[53] Giraldo L., Moreno-Pirajan J.C. (2014). Activated Carbon Prepared From Orange Peels Coated With Titanium Oxide Nanoparticles: Characterization and Applications in the Decomposition of NOx. Orient. J. Chem..

[54] Liu Q.-S., Zheng T., Wang P., Guo L. (2010). Preparation and characterization of activated carbon from bamboo by microwave-induced phosphoric acid activation. Ind. Crops Prod..

[55] Ip A., Barford J., McKay G. (2008). Production and comparison of high surface area bamboo derived active carbons. Bioresour. Technol..

[56] Chang J., Gao Z., Wang X., Wu D., Xu F., Wang X., Guo Y., Jiang K. (2015). Activated porous carbon prepared from paulownia flower for high performance supercapacitor electrodes. Electrochimica Acta.

[57] Zhu X.-L., Wang P.-Y., Peng C., Yang J., Yan X.-B. (2014). Activated carbon produced from paulownia sawdust for high-performance CO2 sorbents. Chin. Chem. Lett..

[58] Yorgun S., Vural N., Demiral H. (2009). Preparation of high-surface area activated carbons from Paulownia wood by ZnCl_2_ activation. Microporous Mesoporous Mater..

[59] Velásquez-Cock J., Castro C., Gañán P., Osorio M., Putaux J.-L., Serpa A., Zuluaga R. (2016). Influence of the maturation time on the physico-chemical properties of nanocellulose and associated constituents isolated from pseudostems of banana plant c.v. Valery. Ind. Crop. Prod..

[60] Valledor L., Guerrero S., García-Campa L., Meijón M. (2021). Proteometabolomic characterization of apical bud maturation in Pinus pinaster. Tree Physiol..

[61] González J.F., Román S., González-García C.M., Valente Nabais J.M., Ortiz A.L. (2009). Porosity Development in Activated Carbons Prepared from Walnut Shells by Carbon Dioxide or Steam Activation. Ind. Eng. Chem. Res..

[62] Wigmans T. (1989). Industrial aspects of production and use of activated carbons. Carbon.

[63] Román S., González J.F., González-García C.M., Zamora F. (2008). Control of pore development during CO2 and steam activation of olive stones. Fuel Process. Technol..

[64] Li H., Chen J., Zhang W., Zhan H., Yang C.H.Z., Peng H., Leng L. (2023). Machine-learning-aided thermochemical treatment of biomass: A review. Biofuel Res. J..

[65] Panahi H.K.S., Dehhaghi M., Ok Y.S., Nizami A.-S., Khoshnevisan B., Mussatto S.I., Aghbashlo M., Tabatabaei M., Lam S.S. (2020). A comprehensive review of engineered biochar: Production, characteristics, and environmental applications. J. Clean. Prod..

[66] Aghbashlo M., Khounani Z., Hosseinzadeh-Bandbafha H., Gupta V.K., Amiri H., Lam S.S., Morosuk T., Tabatabaei M. (2021). Exergoenvironmental analysis of bioenergy systems: A comprehensive review. Renew. Sustain. Energy Rev..

[67] Abdelaziz G.B., El-Said E.M., Bedair A.G., Sharshir S.W., Kabeel A., Elsaid A.M. (2021). Experimental study of activated carbon as a porous absorber in solar desalination with environmental, exergy, and economic analysis. Process. Saf. Environ. Prot..

[68] Aghbashlo M., Hosseinzadeh-Bandbafha H., Shahbeik H., Tabatabaei M. (2022). The role of sustainability assessment tools in realizing bioenergy and bioproduct systems. Biofuel Res. J..

